# The physician-scientist: looking back, looking forward

**DOI:** 10.1172/jci.insight.192637

**Published:** 2025-04-08

**Authors:** Gary Koretzky

**Affiliations:** Cornell University, Ithaca, New York, USA.

## Abstract

Efforts dating back to the 1950s have ​sought to formalize educating physician-scientists, individuals trained in both science and medicine and who devote the bulk of their professional time to investigative work. The return on investment has been huge, because, as a group, these individuals have made outsized contributions to advancing human health. However, efforts at supporting the physician-scientist career path have been accompanied by repeated concerns regarding the lack of intentional support needed to sustain trainees and faculty. This Perspective reviews the history of the career path, highlighting both opportunities and challenges, and offers seven recommendations with the potential to both promote its vitally and reinvigorate its future at all its stages.

Following World War II, scientific research in the United States became a national priority, with large financial commitments to support investigators and to train the next generation of scientists. National centers of research grew exponentially, and research efforts supported by the government and carried out at academic institutions and the private sector flourished. A scientific infrastructure was born, in large part prompted by the visionary leadership of Vannevar Bush, an engineer and advisor to President Franklin Roosevelt (1). Investments were made in all scientific domains, and biomedical research became a major recipient of these commitments. While the major emphasis on building the scientific workforce initially focused on PhD scientists, there was increasing awareness of the unique contributions that had been and could continue to be made by physician-scientists, individuals with combined robust medical and scientific training. Formal recognition of the roles that could be played by this cadre of investigators to improve the health of the population was soon to come as research in biomedicine became firmly established through the NIH.

James Shannon, the NIH director from the mid-1950s to the mid-1960s, is credited with putting many of the programs into place that recognized the value and unique contributions of physician-scientists (2). While the definition of exactly who should be considered a physician-scientist evolved (and continues to evolve), for the purpose of this Perspective piece, we will consider physician-scientists as those individuals trained in medicine who devote the bulk of their professional time to investigative work. This might be at the bench, through clinical investigation, or analyzing the huge data sets that are becoming available to inform the spectrum of science from the most fundamental principles to devising new approaches for patient care. Under Shannon’s leadership, the NIH took on the mantle of supporting the work of current physician-scientists (both through NIH intramural and extramural programs) and envisioning ways to promote the career path for the next generation.

As readers of *JCI Insight* know, although physician-scientists have had an outsized impact on advances in medicine, the career path has had twists, turns, and bumps. For this article, I will briefly review what it meant to be a physician-scientist in the so-called “prescientific era of medicine” and then hone in on aspects of the physician-scientist career path in the United States. This Perspective piece will remind readers of challenges faced by physician-scientists that have been articulated by eminent academic leaders over the past fifty years. Additionally, it will provide suggestions for consideration by individual physician-scientists, institutions, and the American Society for Clinical Investigation (ASCI), as we work collectively to support the future success of the talented individuals committed to this career path.

## The “pre-Flexnerian” era

For centuries, physicians have taken on multiple roles as they pursued their profession. First and most importantly, they supported the health and well-being of their patients using tools available to them at that time. However, physicians were also called upon to do more — often serving as public health officials advising communities on best practices, especially in times of disease outbreaks. Some devoted their careers to the education of the next generation of physicians, originally through an apprenticeship model and then through a more formal educational process. Always, however, there were individuals trained in medicine who devoted their careers to the generation of new knowledge, confident that it would only be through discovery that care for patients would improve.

Although the practice of and training for medicine is generally considered “prescientific” before the early twentieth century, some of the most important advances in biology through the ages — from the description of how blood circulates in the body by William Harvey in 1628 (3), to the discovery of the smallpox vaccine by Edward Jenner in 1796 (4), to the work of Robert Koch who envisioned a test of the germ theory of infectious diseases in 1876 (5) — were spearheaded by clinicians. Additionally, professional societies such as the Royal College of Physicians in the United Kingdom, chartered in 1518 (6), were created to formalize a process to promote standards of medical practice and to bring physicians together to discuss innovations that could be applied to patient care. Cadres of physician-scientists were not merely interested in the scientific basis of physiology and disease — many became international leaders in their scientific disciplines, mentoring others and serving to guide research directions of their institutions. The enormous impact of physician-scientists on advancing clinical practice is difficult to quantify, but perhaps one statistic is notable. In the first 25 years that Nobel Prizes in Medicine were awarded, there were a total of 23 awards (in some years there were two awards and in others none), of which 15 laureates (65%) were physicians (7). Of course, physician-scientists did not do their work in a vacuum. They often collaborated with nonphysician colleagues trained in the principles of science, a tradition that continues to this day, promoting synergies as different perspectives, experiences, and funds of knowledge are brought together.

Before the early 1900s, training in most medical colleges did not emphasize the scientific underpinnings of medicine, and the choice of the physician-scientist career path was largely ad hoc. Even so, there were some medical colleges in Europe and the United States that recognized the value of discovery and supported select faculty in their research efforts. In the middle of the nineteenth century, some academic leaders were beginning to articulate the unique importance of physician-scientists. As quoted in the 2014 Physician-Scientist Workforce Working Group Report (8), commissioned by the NIH (and referred to in more depth below), Claude Bernard, the mid-nineteenth century French physiologist working at the Sorbonne and best known for describing the milieu intérieur, remarked in 1865, “In a word, I consider hospitals only as the entrance to scientific medicine; they are the first field of observation which a physician enters; but the true sanctuary of medical science is a laboratory; only there can he seek explanations of life in the normal and pathological states by means of experimental analysis.” This description of the raison d’etre for physician-scientists continues to resonate for many today.

## Applying science to medicine

The broad notion of formalizing the relationship between science and medicine (and making it possible to define the career path of physician-scientists) began to develop in the United States at the end of the nineteenth century. In 1885, Willian Osler and six of his colleagues formed the Association of American Physicians (AAP), with the goal of “the advancement of scientific and practical medicine” (9). In 1905, the relatively young Association of Medical Colleges (AAMC) adopted a blueprint for a formalized medical school curriculum, including instruction in basic science as well as clinical medicine (10). Three years later, the ASCI was founded following a meeting in Atlantic City of a group of younger physician-scientists who had not yet met the criteria for membership in the AAP and believed that a more inclusive society of physician investigators would provide an excellent forum for reporting research findings and advancing practice (11). Then, in 1910, the Flexner Report, commissioned by the Carnegie Institute and authored by Abraham Flexner (an educator, not a physician or scientist) expanded on earlier work of the AAMC to advocate for institutionalizing and standardizing medical training, with an emphasis on both fundamental science and the development of clinical skills (12). The Flexner Report is widely credited with creating an approach to medical education that continues to this day (although, of course, the details of how instruction is delivered and the extent of basic science in medical school curricula continue to be debated).

With this change in focus for medical education, academic medical centers in the United States embraced the notion that among their institutional missions was the generation of new knowledge that could be applied to patient care. Medical colleges began to expect that, in addition to providing care to patients and teaching students, faculty should contribute to the generation of this new knowledge. At the federal governmental level, there were a series of steps taken to authorize a sustainable research infrastructure and to provide financial support for research outside of government facilities. Following Vannevar Bush’s report, NIH was the federal institution given the responsibility for organizing and supporting biomedical research.

In the 1950s, NIH leadership recognized that development of a cadre of physician-scientists at American medical colleges could no longer be ad hoc. Indeed, it would be necessary to develop programs with the explicit intent of supporting individuals as they considered and then trained for this career path. The first support effort began in the mid-1950s with the Associate Training Program (ATP) (13), a competitive two-to-three-year fellowship that brought recent MD graduates to the NIH Bethesda campus for intensive research training under the guidance of an intramural investigator. For many of the associates, the NIH experience was their first foray into research.

The NIH ATP grew considerably in the 1960s during the height of the Vietnam War. During those years, male graduates of medical schools were subject to the military draft. Some physicians were posted to Vietnam, others to military hospitals in the United States and abroad, and others successfully competed for positions in the NIH ATP, which was run under the auspices of the Public Health Service and met the service requirement of enrollees. As the war continued, the number of ATP applicants increased (there were 3,075 such applicants between 1965 and 1975), and the program had its pick of outstanding candidates. Some graduates of the program continued their careers at the NIH, while others spread out among academic medical centers across the country, often eventually taking on leadership roles at schools of medicine. More than 75% stayed in academia for their first position after completion of the program, and most participants continued performing investigative work consistent with the career path of physician-scientists (13).

The NIH ATP focused on immersing individuals in science after completion of medical school. In the late 1950s and early 1960s, some US medical schools began experimenting with the idea of combining medical and scientific training earlier on the career path through programs designed to award graduates both MD and PhD degrees (14). The idea was that there were some individuals who, although just starting medical school, hoped that rigorous investigation would play a central role in their careers and that earlier training in foundational science would accelerate progress toward an independent career. These programs did well (graduates appeared to follow the intended career trajectory), and the concept was formalized and institutionalized through the NIH Medical Scientist Training Program (MSTP), a grant program awarded to institutions that could demonstrate the infrastructure and commitment to shepherd a select group of students through the rigors of medical and scientific training (14).

The first MSTP grants were awarded to three medical schools in 1964. The program has continued uninterrupted since then, and at the end of 2024, there were 58 funded programs, two dedicated to Doctor of Veterinary Medicine/PhD (DVM/PhD) candidates, two joint programs supporting MD/PhD and DVM/PhD candidates, and 54 programs available for MD/PhD candidates (15). The MSTP grants do not support all the costs of the programs. There is a required institutional financial commitment, and students are typically supported through mentors’ grants during the research phase of their training. This cost sharing enables institutions to train a greater number of students compared with the number of slots that are provided through the NIH grant. Moreover, a substantial number of medical schools offer MD/PhD training, even without an MSTP grant. Today, there are between 550 and 600 graduates from US MD/PhD programs each year (16). However, barely a decade after the first MSTP grant was awarded, rumblings began in academic communities, as challenges to the physician-scientist career path became evident.

## Viability concerns for the physician-scientist career path

Nearly fifty years ago, although by many measures, physician-scientists were thriving (discoveries were being made, and physician-scientists were playing leadership roles at premier academic institutions, leading pharmaceutical companies, and serving as directors of NIH Institutes and Centers), senior leaders in academic medicine began to sound an alarm. The first high-profile publication (17) to this effect was entitled, “The Clinical Investigator as an Endangered Species,” and it appeared as a special article in the *New England Journal of Medicine* in late 1979, authored by James Wyngaarden, then Chair of Medicine at Duke and soon-to-be director of the NIH. In this article, Wyngaarden noted a decline in interest on the part of physicians headed toward investigative careers. This was occurring at a time when the number of PhD scientists was expanding, and Wyngaarden noted that training grant slots for programs initially designated for physicians were being left unfilled. Wyngaarden also noted the long-time horizon for combined medical and scientific training and predicted that the trends he was observing (decreased interest in the physician-scientist career path) would only accelerate into the future.

Following Wyngaarden’s paper, similar perspective pieces were published by academic medicine leaders with regularity. Readers of this Perspective are likely aware of several — Joe Goldstein’s 1986 ASCI presidential address describing the “Paralyzed Academic Investigator’s Disease Syndrome” (18), the 1997 Joe Goldstein and Mike Brown *JCI* Perspective on the “bewitched, bothered, and bewildered — but still beloved” clinical investigator (19), the Leon Rosenberg 1999 *JCI* report describing physician-scientists as the “essential — and fragile — link in the medical research chain” (20), and a series of papers in the early 2000s by Stephen Archer telling stories (and describing some of the elements required for their successes) of some of the most notable physician-scientists of that time and the pressures the current generation was experiencing (21).

Many of these papers had similar themes. They detailed the outsized impact physician-scientists have had on clinically important discoveries, and, despite the fact that many physician-scientists considered their career amazingly rewarding, the papers noted that, even highly successful physician-scientists faced unique challenges as they established and cemented their careers. The papers cite several such factors, including the long period of training and navigating the complex world of competing for research grants and the simultaneous pressure to leave the laboratory for the clinic, as institutions faced financial pressures that required increasing clinical revenue. Work-life balance concerns were highlighted, as the most intensive period of physician-scientist training occurs at just the same time many are hoping to start families, a tension that is even greater for female physician-scientists. The papers also reported on financial pressures, especially for trainees who were not graduates of MSTPs — who receive tuition waivers and stipend support during training, diminishing debt. In contrast, trainees who were not MSTP graduates who choose research intensive fellowships often face significant debt, making a delay in obtaining competitive compensation personally problematic. Each of the papers urged funding agencies and institutions to identify ways to support physician-scientists through their training and the early stages of their independent careers. Many also emphasized the importance of trainees having mentors to help them navigate their career path.

These concerns did not fall on deaf ears. The NIH created a series of new programs to support early-stage investigators, both those with PhD and those with MD training. Because one of the drivers identified by those leaving the physician-scientist career path was educational debt, a loan repayment program was established (in large part with leadership from the ASCI and the Federation of American Societies for Experimental Biology) to diminish the financial burden on trainees pursuing research training. Private philanthropies, such as the Howard Hughes Medical Institute and later the Burroughs Wellcome Fund, created programs specifically to support physician-scientists; however, many of these programs have since been terminated. Medical specialty societies similarly created programs to support physician-scientists in their areas of specialization; however, these programs varied markedly from society to society. Top tier academic medical centers established research residency programs to recruit and support house staff committed to scientific training. Nearly all these programs included, to some extent, financial support during the research-intensive years of training as well as mentoring opportunities to help trainees learn to navigate the complexities of the career path. However, even with these efforts, data obtained by the AAMC revealed that medical school graduates were less and less likely to list research as a primary goal for their postgraduate careers, and concerns about the viability of the physician-scientist career path mounted.

As the community continued discussions about ways to maintain physician-scientist vitality, one important issue received less attention than it deserved. Over the last 50 years, tremendous (and successful) efforts were made to ensure parity among male and female medical school matriculants. In the 1970s, it was typical for female students to represent less than a quarter of a medical school class. This changed gradually until in 2019 there were more first-year female medical students compared with male medical students in American medical schools (22). However, the story for physician-scientist trainees and faculty is quite different, as male individuals still significantly outnumber female individuals at every step in the career pathway. Moreover, the discrepancy increases as individuals advance in their career. This was brought to the community’s attention in a 2008 *Science* paper by Timothy Ley and Barton Hamilton (23). Subsequent surveys show that this is multifactorial (gender bias, lack of role models, care-giving responsibilities), all considerations that can be remedied. One promising sign is a recent increase in female MSTP matriculants (although still not at parity); however, discrepancies continue to increase at every subsequent stage of the career path. This is a huge loss in many ways. Not only do female individuals have less opportunity to participate in a rewarding and impactful career, but half the population that could contribute to the next generation of discovery is underrepresented.

In 2012, the NIH Biomedical Research Workforce Working Group (a committee charged with examining career trends for PhD and MD scientists) reported that while the needs and concerns of physician-scientists overlapped with those of the much larger PhD scientist workforce, there were significant, distinct issues between groups that needed attention. The working group recommended that a separate committee, dedicated to examining those specifically affecting the physician-scientist workforce, assess both the opportunities and challenges this group experiences. Accordingly, Francis Collins, the NIH director at the time, commissioned a blue-ribbon panel. This panel reported out its findings in 2014 (8).

The report was comprehensive and data rich. It told the community much of what was already known — that without attention, the career path of physician-scientists was imperiled. Many of the concerns articulated in publications in preceding years were repeated and backed up with data showing trends over time. As a working group commissioned by the NIH, the recommendations focused on what the NIH could contribute to reverse the situation. Nine top-line recommendations were made, including continued and enhanced support of MSTPs, development of grant programs to further assist training physician-scientists as they established their independent research programs, decreasing debt burden by enhancing the loan repayment program, and establishment of programs to increase the diversity of the physician-scientist workforce and to diminish barriers that may affect specific subgroups of trainees. The working group also recommended that there be requests for proposals for projects that would explore ways to shorten the time of training for physician-scientists and that the NIH regularly assess the state of the workforce, to review trends, but also to assess the impact of newly established programs.

The report was widely appreciated, and several of the recommendations turned into action. However, a 2022 a report in the *FASEB Journal*, authored by Howard Garrison and Timothy Ley, continued to voice concerns (24). This publication reviewed the state of the physician-scientist workforce during the decade from 2011 through 2020, particularly in light of the 2014 NIH report. They found some hopeful signs — increasing numbers of matriculants into MD/PhD programs and an increased number of medical students doing research projects. They also found that efforts to provide early-stage physician-scientists with R01 support seemed to have had a positive impact. However, the authors were alarmed to find that despite redoubled efforts on the part of many to reverse the trends, the number of medical school graduates indicating that they would pursue research continued to decline in AAMC survey responses. Perhaps related to this, applicants for the loan repayment program decreased over this period. Another worrisome indicator was that the age when physician-scientists were awarded their first R01 grant continued to increase, suggesting that, although decreasing the time to completion of training was on the minds of many, inroads toward this issue had not yet been accomplished.

Clearly, although physician-scientists have been instrumental in advancing biomedical science, studies are compelling that many talented individuals with enormous potential to succeed in this career find its complexity daunting. This starts with the length and intensity of training but continues as individuals pursue their careers at every stage. While the rewards are huge, we are still losing creative and idealistic individuals who should make up the next generation. It is incumbent on us to identify the roadblocks and then collectively make changes to ease the path for those following us.

## Suggestions for consideration by individuals, institutions, and the ASCI

With the continued commitment from the ASCI and *JCI Insight*, it is hoped that this Perspective piece will not be the end of a dialogue but a beginning. I will conclude this piece by providing seven thoughts for consideration. Two are directed toward my peers, physician-scientists committed to their own work and the success of the next generation; three are directed toward the institutions where we work, including academic medical centers and the NIH; and two are directed toward the ASCI, as the leading organization committed to the success of those committed to the physician-scientist career path. In making these recommendations, I am speaking only for myself, not as a representative of an institution, group, or society. I hope that readers will find these useful.

## For consideration by individuals

### Communicate what you do and why you do it to the public.

Scientists have always faced the challenge that the nonscientific community has difficulty understanding how scientists go about their craft. At times, the mystique of the scientific process worked in favor of scientists, as the public assumed good intentions and that even if they did not appreciate the nuances of how science moved forward, they respected individuals committed to investigative work. There have been other times where this was decidedly not the case and efforts of scientists were either ignored or derided. Unfortunately, we are living in a world today where scientists are being increasingly required to justify what they do to a skeptical public. There is an underlying feeling of distrust that, at times, moves into the realm of antipathy. Regardless of the source of these feelings, it is the obligation of the scientific community to be more forthcoming so that those not immersed in science can better appreciate the process of discovery. This is important because it is the public that funds investigation and it is their right to understand what scientists do. Beyond that, it is our obligation as members of society to do what we can to promote evidence-based decision making.

Communication about complex issues, like the process of scientific investigation, is an art. Individuals will have to consider the best way they can participate — perhaps through public lectures at schools, community centers, or libraries, or perhaps through writing. Given that the ability to communicate science broadly is not intuitive but a skill to be learned, one suggestion is to find ways to incorporate scientific communication skill building into our training programs so that the next generation of physician-scientists will have experience and more comfort in talking to wider audiences.

### Mentoring the next generation.

I will always remember the individuals who took time and effort to help me launch my career. These included not only my official mentors, but also individuals who were committed to advising the next generation, even if they had no direct responsibility for an individual’s training or success. I expect that all readers of this Perspective have similar memories. Being that mentor is valuable, not only to our trainees, but also to us, as helping more junior colleagues learn how to navigate the complex world of academic medicine is intrinsically gratifying. In this time of uncertainty, mentoring will take on even greater importance, and being open and available to our trainees is key.

## For consideration by institutions

### Create an infrastructure dedicated to the support of physician-scientists.

Support for faculty at today’s academic medical centers has become increasingly complex. Whereas a generation ago expectations of nearly all faculty — clinical service, teaching, and scholarship — were similar (albeit with faculty placing differential emphasis on one or several of these missions), today’s faculty are segregated into different tracks with different professional expectations. Physician-scientists typically make up the minority of faculty at today’s medical colleges, and, as detailed in this Perspective, there are unique pressures on them as they navigate their careers. In recognition of this, some institutions have created an infrastructure for physician-scientist support, the form of which varies from institution to institution. Ideally, such an infrastructure would have dedicated resources to advocate for physician-scientists at the highest leadership level. It should provide opportunities for vertical integration, creating a community of physician-scientists spanning medical student trainees to senior faculty, so that individuals at different stages of their careers can learn from each other. There should also be opportunities for horizontal integration so physician-scientists across disciplines become a supportive community. Having a robust infrastructure provides a means to formalize support mechanisms and mentoring programs.

As institutions consider ways to support the physician-scientist community, we must be mindful that our definition of what it means to be a physician-scientist must evolve as science moves forward. Moreover, we must become more creative about how best to nurture talented trainees as they pursue their career aspirations. The MSTP model, where individuals spend 7–8 years simultaneously to pursue MD and PhD degrees, served our community well; however, it is not the only way to prepare for a career as a physician and investigator. It is incumbent on institutions to continue to support and expand the range of programs capable of providing the next generation opportunities to pursue clinical investigation as their primary professional activity.

Creating a robust infrastructure to support the career path would also help provide an opportunity to monitor progress. Knowing in advance what success would look like with careful collection and analysis of outcomes would help physician-scientist support offices tweak programs to be more effective. Moreover, having a formalized infrastructure would provide institutional memory to maximize future efforts. It is hoped that institutions that create such infrastructures will share ideas with peers. While what works at one institution may not be appropriate for another, collectively we may define best practices to share.

### Regularly assess opportunities and challenges related to the physician-scientist workforce.

The 2014 NIH physician-scientist workforce report provided essential information to the physician-scientist community and made recommendations with potential for positive impact. Notably, though, this report reflected the status of the career path at one point in time. In fact, the report itself recommended that, to be most effective, there should be regular evaluations of the opportunities and challenges facing the workforce (8). Following through on this recommendation and expanding the focus of periodic evaluations would add great value to the conversation about maintaining the health of the career path.

It is suggested that the NIH commission periodic reviews, perhaps every five years. This will allow the community to examine trends and evaluate the success of initiatives that are piloted. It is also suggested that these reviews be broader in scope, evaluating institutional efforts and programs sponsored by private foundations and medical specialty societies to gain more insight into efforts that seem to be bearing fruit.

### Address the gender gap among physician-scientists at all career stages.

As noted above, the huge disparity in male versus female medical school matriculants has disappeared, yet there remains a large gap when the number of female compared with male physician-scientist trainees and faculty are considered. It is urgently recommended that institutions take proactive steps to remedy this situation. While there are many factors responsible for these discrepancies, medical schools have solved the problem. It is incumbent on the physician-scientist community to do the same. It is suggested further that trends be tracked, and when progress is made by some institutions, best practices should be shared.

## For consideration by the ASCI

### Be a clearinghouse of best practices.

The many academic institutions around the country may be considered laboratories, each developing their unique approaches to support physician-scientists. As the most prestigious society committed to this goal, the ASCI has the potential to become the repository and communicator of best practices developed by colleagues nationwide. This could be accomplished by the creation of a standing committee of ASCI members, whose charge would be to gather information from academic centers, the NIH, foundations, and medical specialty societies on programs they have established, the metrics for success that they employ, and their outcomes. The ASCI can then become the one-stop clearinghouse for best practices from which colleagues could learn. This committee should be integrated with the periodic reviews conducted by the NIH evaluating challenges and opportunities facing the workforce. The committee could share information it gained through sponsoring a session at the ASCI annual meeting.

### Support projects assessing the return on investment of the physician-scientist career path.

Over the past 50 years, most publications raising the alarm about the future of physician-scientists assume (or use anecdotal data to support the notion) that physician-scientists provide unique value to the biomedical research enterprise. Although I would venture that nearly everyone reading this Perspective wholeheartedly agrees with this premise, I suggest that an argument for additional effort and investment to bolster the career path would be more compelling if the value of the physician-scientist career path was rigorously examined. This final suggestion for consideration by the ASCI is that our society support studies that examine, from all vantage points, that return on investment. The notion would be for the ASCI to issue a request for applications that would be competitively reviewed with resources provided for careful analysis, the results of which could be collected and shared as Physician-scientist development papers at *JCI Insight*. Awards should be given to investigators or groups of investigators who propose projects dependent on the skills physician-scientists use in their everyday lives — generating hypotheses that are put to rigorous testing and thoughtful evaluation of the data. The results of these projects would ideally inform suggestions for how best to support the next generation of physician-scientists.

## Version 1 04/08/2025

Electronic publication
